# Historical Cohort Study on the Factors Affecting Blood Pressure in Workers of Polyacryl Iran Corporation Using Bayesian Multilevel Modeling with Skew T Distribution

**DOI:** 10.5812/ircmj.10930

**Published:** 2013-05-05

**Authors:** Mohammad Gholami Fesharaki, Anoshirvan Kazemnejad, Farid Zayeri, Javad Sanati, Hamed Akbari

**Affiliations:** 1 Biostatistics Department, Faculty of Medical Sciences, Tarbiat Modares University, Tehran, IR Iran; 2 Proteomics Research Center, Faculty of Paramedical Sciences, Shahid Beheshti University of Medical Sciences, Tehran, IR Iran; 3 Occupational Health Center, Polyacryle Company, Tehran, IR Iran; 4 Health Research Center, Baqiyatallah University of Medical Sciences, Tehran, IR Iran

**Keywords:** Blood Pressure, Cohort Studies, Multilevel Analysis

## Abstract

**Background:**

Hypertension is considered as a major public health problem in most countries due to its association with ischemic heart disease which causes cerebrovascular disease and death.

**Objectives:**

The purpose of the present study was to study factors affecting Blood Pressure (BP).

**Patients and Methods:**

The data were extracted from annual observation of the workers who worked in Polyacryl Iran Corporation (PIC) between 1998 and 2010. In this research, we assessed the effect of Body Mass Index (BMI), age, sex, job status, marital status, job schedule type, and education level on Systolic Blood Pressure (SBP) and Diastolic Blood Pressure (DBP) using Bayesian multilevel modeling with skew t distribution using WinBUGS software.

**Results:**

Totally 3965 persons participated in this study, 75(1.9%) female and 3890 (98.1%) male. In this study age, sex, BMI, job status, marital status, and education level had statistical association with SBP. The result for DBP was similar to SBP except the education level which had no statistical association.

**Conclusions:**

Treating obesity, increasing physical activity and quality of married life are proposed as practical solutions to reduce BP.

## 1. Background

Blood Pressure (BP) is the force of blood pushing against the walls of the arteries as the heart pumps blood (1). If this pressure rises and stays high over time, is called hypertension. Hypertension is a chronic disorder which imposes heavy treatment and care costs (2) and it is a serious condition which can lead to coronary heart disease (3), heart failure (4), stroke (5), devastating effect of other cardiovascular risk factors (like dyslipidemia, smoking, diabetes and obesity) (6), and other health problems (1, 2, 7). The overall prevalence of hypertension is 17.8% in Iran (8), and its prevalence in the age group 30-55 and more than 55 are 23% and 50%, respectively (9). Previous studies have shown the association of various factors like obesity (10-12), age (13), gender (12, 14-16), marital status (17), quality of married life (18), smoking and being exposed to cigarette smoke (19, 20), loud noise at work place (21), workload (22), stress (23, 24), diet and physical activity (25), shift work (26), gaining weight (11, 12) with BP.

## 2. Objectives

Considering the importance of BP and since different studies have reported contradicting findings regarding factors affecting it, we decided to perform this historical cohort study to investigate the factors affecting BP using Bayesian multilevel modeling with skew t distribution.

## 3. Patients and Methods

This historical cohort study enrolled employed workers of Polyacryl Iran Corporation (PIC) in the Esfahan city from 1998 to 2010 with average repetition of 6.5 times and mean interval of 2 years using census sampling method. PIC produces polyester yarn and fiber and is the only producer of acrylic fiber in Iran country. In this study, inclusion criteria were official employment and attending annual health examinations between 1998 and 2010, and exclusion criteria were treated previously for hypertension, retirement, death or dismissal. During this period, 22 018 employees received the health examination. Based on the mentioned criteria, 4 525 observations were excluded from the study. This study was approved by the ethical committee of the medical school of Tarbiat Modares University, issued on 05.11.2011. Its registration number is 5271065. In this study, BP of both arms was measured in the sitting position after 5 minutes rest using a calibrated mercury sphygmomanometer. Also, weight and height were measured by a physician using calibrated equipment. In this study the variable shift schedule was categorized as routine rotating shifts (2 morning shifts, 2 evening shifts, 2 night shifts and 2 days off) and weekly rotating shifts (3 morning shifts, 3 evening shifts, and one day off every two weeks, Fridays always off). Regular day workers worked from morning to evening on weekdays, and had Thursdays and Fridays off. The morning, evening, and night shifts began at 7 AM, 3 PM, and 11 PM, respectively. Day workers worked from 7 AM to 3 PM on weekdays, Thursdays and Fridays off.

### 3.1. Data Analysis

In this study we used Bayesian multilevel modeling with skew t distribution. Multilevel modeling is a useful method for analyzing correlated and longitudinal data (27). Routine assumption in multilevel modeling considers normal distribution for random error terms. Unfortunately, this assumption may be unrealistic in some situations, since the normal distribution is not able to reflect all features of error terms appropriately and therefore inferences about the model parameters may be misleading. To overcome this problem we considered skew t-distribution (28) instead of normal distribution for random error terms and then used Bayesian approach with vague prior distributions (assuming an exponential distribution with lambda 0.1 for degree of freedom, a normal distribution with mean 0 and variance 100 for the beta parameters, and postulating a gamma distribution with parameters alpha = 0.001 and beta = 0.001 for the variance parameters). Then we used WinBUGS software for Bayesian analysis. Results were based on every 100 draw from a Markov Chain Monte Carlo (MCMC) chain of length 11,000 with a burn-in of 1000. This proved more than enough for convergence, and much shorter runs led to virtually identical results. In this paper we also checked normal distribution for random error terms with Kolmogorov–Smirnov test.

## 4. Results

During 1998 to 2010 3965 workers and employees underwent the annual evaluation by the health and safety executive office of PIC. The mean follow up time of the workers was 6.5 repetitions. The demographical characteristics of the participants at their first health examination are presented in Table 1. According to this table, most subjects were male, married, educated as diploma or lower diploma, and they were day workers and blue-collar workers. Tables 2 and 3 show the summary of MCMC beta, their standard deviation, standard errors and statistical significance using Bayesian multilevel modeling with skew t distribution for the association between predictor variables on SBP and DBP respectively. According to the results, age, BMI, sex, job status, marital status and education level had statistical relation with SBP. The result for DBP was similar to SBP, except the education level which had no statistical association. In addition, significant parameter of Bayesian multilevel modeling with skew t distribution (within and between subject variance, skewness parameter and T degree of freedom) shows that such model is a convenient model for our historical data.

**Table 1.** The Demographicaland Baseline Characteristics Baseline of the Workers at Their First Health Examination

Continuous Variables	
**Variables **	Median (Mean ± SD)
**Age, y**	24.64 (25.61 ± 4.16)
**Work experience, y **	15.39 (12.55 ± 7.99)
**BMI^a^, kg/m^2^**	25.58 (25.65 ± 3.43)
**DBP^a^, mm Hg**	77.50 (77.62 ± 7.68)
**SBP^a^, mm Hg**	120 (120.92 ± 11.23)
**Categorical Variables**	
**Variables**	No. (%), (n = 3965)
**Sex**	
Female	75 (1.9)
Male	3890 (98.1)
**Marriage**	
Married	829 (20.9)
Single	3136 (79.1)
**Education**	
Diploma or lower diploma	1562 (39.4)
Associated degree	1427 (36)
Bachelor or upper	976 (24.6)
**Shift Schedule**	
Weekly rotating	294 (7.4)
Routine rotating	1776 (44.8)
**Type of Job**	
Day workers	1895 (47.8)
Blue-collar worker	3656 (92.2)
White-collar worker	309 (7.8)

^a^Abbreviations: BMI, body mass index; DBP, diastolic blood pressure; SBP, systolic blood pressure

**Table 2.** Bayesian Multilevel With Skew T Regression Results for Assessing the Effect of Predictor Variables on Systolic Blood Pressure

Variables	MCMC ^a^ Beta	MCMC SD ^a^	MCMC SE ^a^	MCMC 95% CI ^a^		P value
				Lower	Upper	
**Age, y**						
**BMI ^a^, kg/m^2^**	0.42	0.018	0.002	0.42	0.45	< 0.001
**Education**	0.67	0.036	0.003	0.59	0.75	< 0.001
Bachelor or upper	-1.65	0.509	0.032	-2.66		
Associated degree	-0.83	0.385	0.023	-1.60	-0.69	0.001
Diploma and lower diploma	Reference category				-0.08	0.016
**Shift schedule**						
Weeklyrotating shift workers	0.35	0.648	0.030	-0.91	1.61	0.295
Routine rotating shiftworkers	0.33	0.378	0.023	-0.370	1.11	0.191
**Type of job**						
Day workers	Reference category					
Blue-collar worker	-1.17	0.67	0.061	-2.6	0.11	0.040
White-collar worker	Reference category					
**Marriage**						
Single	7.28	0.551	0.049	6.33	8.53	< 0.001
Married	Reference category					
**Sex**						
Male	10.60	1.135	0.110	8.41	12.87	< 0.001
Female	Reference category					
**Within subject variance**	5.65	0.419	0.038	4.83	5.65	< 0.001
**Between subjects variance**	67.05	2.367	0.071	67.03	71.79	< 0.001
**Skewness** ** parameter**	1.14	0.075	0.006	1.01	1.13	< 0.001
**T degree of freedom **	7.50	0.49	0.042	0.04	6.62	< 0.001

^a^Abbreviations: BMI, body mass index; CI, confidence interval; MCMC, markov chain monte carlo; SD, standard deviation; SE, standard error

**Table 3.** Bayesian Multilevel With Skew T Regression Results for Assessing the Effect of Predictor Variables on Diastolic Blood Pressure

Variables	MCMC ^a^ Beta		MCMC SD ^a^	MCMC SE ^a^	MCMC 95% CI ^a^		P value
					Lower	Upper	
**Age, y,**	0.46		0.013	0.001	0.43	0.48	< 0.001
**BMI ^a^, kg/m^2^**	0.51		0.03	0.003	0.46	0.56	< 0.001
**Education**							
Bachelor or upper	-0.10		0.36	0.02	-0.80	0.65	0.391
Associated degree	0.18		0.29	0.016	-0.37	0.79	0.267
Diploma and lower diploma	Reference category						
**Shift schedule**							
Weekly rotating shift workers	-0.47	0.44		0.021	-0.47	0.40	0.143
Routine rotating shift workers	-0.42	0.27		0.013	-0.94	0.12	0.060
**Type of job**							
Day workers	Reference category						
Blue-collar worker	-1.25	0.44		0.038	-2.08	-0.30	0.002
White-collar worker	Reference category						
**Marriage**							
Single	6.53	0.35		0.030	5.85	7.21	< 0.001
Married	Reference category						
**Sex**							
Male	9.23	1.01		0.098	7.08	11.66	< 0.001
Female	Reference category						
**Within subject variance**	4.47	0.33		0.031	4.46	5.21	< 0.001
**Between subjects variance**	34.92	1.29		0.049	32.49	37.51	< 0.001
**Skewness parameter**	-1.18	0.07		0.006	-1.32	-1.04	< 0.001
**T degree of freedom**	5.86	0.390		0.034	5.83	6.70	< 0.001

^a^Abbreviations: BMI, Body Mass Index; CI, Confidence Interval; MCMC, Markov Chain Monte Carlo; SD, Standard Deviation; SE, Standard Error

## 5. Discussion

Because of the importance of hypertension, this study was performed to investigate the factors affecting BP in a historical cohort of PIC. In this study, Age showed a positive association with SBP and DBP, Each one-year increase in age elevated SBP and DBP by 0.42 and 0.46 mmHg, respectively. Several cohort and cross sectional studies have confirmed the direct association between BP and aging in different societies (13). BMI also had a direct association with SBP and DBP, indicating that obesity increases SBP and DBP. The direction and significance of this association were congruent with the findings of previous studies. The direct correlation between obesity and BP has been confirmed in several studies (10-12). In our study, each 1-unit increase in BMI elevated SBP and DBP by 0.67 and 0.51 mmHg, respectively. Education also had a significant association only with SBP. Our results showed that individuals with higher levels of education had lower levels of BP, which could be due to higher income and job satisfaction. The results also showed that men had higher BP than women. This difference was 10.60 mmHg for SBP and 9.23 mmHg for DBP. This finding was in line with previous reports (12, 14-16). In our study, single participants showed higher BP compared to married ones. Holt-Lunstad et al. reported that marriage itself did not affect blood pressure, and stated that the quality of married life and couple’s satisfaction was effective in lowering BP in married individuals (18). Moreover, Lipowicz et al. found that men who were never married had higher levels of BP when compared to married men. They used psychological indices (more stress and less social support), nutritional status, and economic situations of living a single life to justify this finding (17). Our findings showed an association between the type of job and BP. Blue-collar workers had lower BP compared to white-collar workers. It is because of more physical activity among blue-collar workers compared to white-collar ones. Respecting the results, our results did not support the association between shift schedule and BP. Studies have reported different and sometimes inconsistent results regarding the association of working on shift work and BP, they have mostly confirmed higher BP in shift workers when compared to day workers (1, 29-31). On the other hand, some studies like our study (32-38) found no significant association between BP and shift. This lack of association can be attributed to the fact that healthier individuals are usually recruited as shift workers while weaker ones are hired as day workers. Moreover, most of the day workers have administrative job and are therefore less active, leading to weight gain (a risk factor of BP elevation). Gholami et al. (39) found a significant increase in body mass index (around 0.78 kg/m2) among day workers compared to weekly rotating shift workers. However, since the effect of shift work on individuals generally depends on the occupation, personal characteristics, workplace environment, and specifications of the shift work (40, 41), this association could be due to other reasons such as the variability of the work time and more income of the shift workers as compared to day workers. In the end, some of the advantages of this study are its longitudinal design, more than 6 years of follow up in average, using a complicated and powerful statistical modeling approach (Bayesian multilevel modeling with skew t distribution), adequate sample size and homogeneity of the study population. However, lacks of access to family history of hypertension in close relatives, inability to evaluate the amount of rest and sleep, income, stress, job satisfaction, and smoking habit as confounding factors are considered some week points of our study. The results of this study demonstrated higher levels of BP in obese workers. We suggest running the SHIMSCO (42) plan to decrease obesity in PIC. Furthermore, considering the effect of marriage, quality of married life and couple’s satisfaction with marriage on lowering BP, counseling sessions are recommended for married couples to enhance the quality of married life. Such counseling sessions can be also held before marriage to help individuals make correct decisions regarding their future life. Moreover, supporting single individuals financially is effective in lowering BP.
